# ﻿Does a citizen science project describe the biogeography of exotic *Aureoboletus projectellus* in Poland? An ethnomycological survey

**DOI:** 10.3897/imafungus.16.166407

**Published:** 2025-10-14

**Authors:** Marcin Pietras, Dominika Robak, Magdalena Terlecka, Łukasz Łuczaj

**Affiliations:** 1 Institute of Dendrology, Polish Academy of Sciences, Parkowa 5, 62-035 Kórnik, Poland Institute of Dendrology, Polish Academy of Sciences Kórnik Poland; 2 Faculty of Biology, Nature Protection, and Sustainable Development, Rzeszów University, ul. Zelwerowicza 4, 35-601 Rzeszów, Poland Rzeszów University Rzeszów Poland

**Keywords:** *
Boletaceae
*, citizen science, ethnogastronomy, ethnomycology, exotic fungi

## Abstract

*Aureoboletus projectellus* is an American *Boletaceae* fungus that appeared on the shores of the Baltic Sea at the beginning of the 21^st^ century. The mushroom was soon gathered by local communities, and fungi enthusiasts travelled from all over Poland to gather this new food item. The aim of our study was to investigate the spread of the invasive *Aureoboletus projectellus* and its use in mycophylic Poland through an interview-based ethnomycological survey (carried out in the field and online). We gathered 274 questionnaires, and recorded many new localities of the species inland, all over the country, far from the original sites of introduction along the Baltic Sea. We have not found any clear correlation between the origin of the collectors coming to hunt it by the Baltic Sea and its localities inland. On the other hand, the interviews conducted as part of the project revealed 56 new localities of *A. projectellus* in Poland. This demonstrates that citizen science initiatives can yield valuable biogeographical and ethnobiological data, even for complex and poorly understood groups of organisms such as fungi.

It seems that the species is already well-established in Poland, and used in dishes similarly to other *Boletaceae* species. Its local names often contain the word ‘American’ or ‘heather’ due to its origin and preferred habitat.

## ﻿Introduction

Exotic organisms are spread to other parts of the world both on purpose and by accident. Often, humans transfer useful foreigners to other regions in order to benefit local economies, or these organisms are culturally important for migrants. Such practises have often caused biological invasions and loss of biodiversity (Sharma et al. 2005; Maema et al. 2016; Kueffer 2017). On the other hand, once exotic plants become common, abundant and invasive, local communities adopt them as food or medicine (Couplan 2015; Taurerauet al. 2021). The level of use of alien plants can, of course, vary, as not all of the uses known in their indigenous range are discovered or accepted in different parts of the world (Branco et al. 2023). In his controversial paper, Moerman (2008) called for more acceptance for invasive organisms, claiming that they would be slowly incorporated into local ecological and economic systems. A new book edited by Applequist (2025) calls for possible full exploitation of the great economic potential of biomass available from invasive plants.

The situation with fungi is slightly different to that of exotic animals and plants. The native ranges of fungi are usually larger than those of plants due to the lightness of their spores. A large number of species have a Paleoarctic or even cosmopolitan distribution. Furthermore, fungi are usually cryptic organisms that form ephemeral fruiting bodies, and they exist mainly as mycelium overgrowing the soil, so the exact mapping of their distribution is more difficult as for plants (Bisby 1943; Litchman 2010; Gladieux et al. 2016). Moreover, species concepts in fungi are unclear, causing a low level of knowledge about fungal distributions on regional and continental scales (Gladieux et al. 2016). Some taxa are first described or known only from an invasive range, and thus fungal invasions are recorded much less frequently than those of plants or animals.

Following the IUCN definition, an invasive taxon is an organism established outside its natural range, where its occurrence threatens native biota, having a proven negative impact on components of native ecosystems. However, for non-pathogenic fungi, such an influence is a challenge to investigate. Thus, according to the definition proposed by Blackburn et al. (2011), the term ‘invasive’ can be used for organisms with self-sustaining populations at significant distances from their native range (Núñez and Dickie 2014). The latter definitions crucially do not require the species to threaten biological diversity. According to IUCN criteria, only a limited number of non-pathogenic fungal taxa can be classified as ‘invasive’. One of the clearest examples is *Tuber indicum*, introduced to Europe from Central Asia (Murat et al. 2008). This edible truffle is confused with the highly valuable European commercial truffle. Furthermore, *T. indicum* competes directly with *T. melanosporum* for space and resources (Murat et al. 2008). Other cases include suilloid fungi and their associated host trees, which can cause significant reductions in soil carbon stocks, accelerate phosphorus mineralization into more labile forms, and trigger short-term shifts toward fast-nutrient cycling decomposition (Chapela et al. 2001). In the Southern Hemisphere, edible *Suillus* taxa frequently co-invade with planted *Pinaceae* species used in forestry (Dickie et al. 2016). However, suilloid fungi are not so frequently used as a source of food in the invasion range (but see Kujawska and Łuczaj 2015). Another example of edible fungus introduction is *Boletus edulis* s.l., a European boletoid taxon spreading in New Zealand, Australia, and South Africa (Hall et al. 1998). All of these species, *Tuber indicum*, *Boletus edulis*, and *Suillus* spp., have been described as examples of invasive edible fungi that have influenced the functioning of the native ecosystem (Dickie et al. 2016).

Boletoid fungi (*Boletaceae*) are one of the most widely eaten families of edible mushrooms in the world, and one of its members, *Boletus edulis*, is often listed as the most preferred mushroom in ethnic cuisines (Boa 2004; Ambrosio et al. 2024). So far, only a few introductions of boletoid fungi have been documented, with one taxon in this group (*Boletus edulis*) recorded outside native range (Vellinga et al. 2009). At the beginning of the 21^th^ century, a North American bolete, *Aureoboletus projectellus* (Murrill) Halling (2015), was recorded along the coast of the Baltic Sea for the first time (Motiejūnaitė et al. 2011; Wrzosek et al. 2017; Banasiak et al. 2019a). The impact of the fungus on local fungal communities in Europe remains unknown. In a previous paper, *A. projectellus* was described as a model organism highly suitable for studying the expansion of macrofungi outside their natural range (Banasiak et al. 2019a). In our study, we used *A. projectellus* for citizen science-based ethnomycological research. The fungus has reliable occurrence data available, occurs frequently in the invasive range, and possesses distinct morphological features, making its identification easy in field conditions, even for non-professional mycologists.

In the era of the ‘global village’, the development of new technologies, and the concentration of life in social media, citizen science projects provide an excellent opportunity for comprehensive mycological research (Heilmann-Clausen et al. 2016). Citizen science data are highly valuable in studies that describe the biogeography and ecology of various organisms (Devictor et al. 2010; César de Sá et al. 2019; Howard et al. 2022; Colombari and Battisti 2023; Aavik et al. 2024). Moreover, such data has recently been used by stakeholders to decide on policies and the management of biological invasions (Price-Jones et al. 2022). In this study, we used an interview-based ethnomycological survey to investigate the spread of exotic *A. projectellus*, assuming that the human vector is crucial for the spread of the fungus in Poland, one of the most mycophilous countries worldwide (Kotowski 2019; Kotowski et al. 2019, 2021). Therefore, this paper aimed to record the interaction of mushroom pickers with the new invasive boletoid species in Poland. Our aim was to answer the following questions:

1. Why do mushroom collectors collect *A. projectellus*?
2. Where do they collect it and/or where do they travel from to do so?
3. What do they do with it?
4. What name do they use for it?
5. Do humans play a role in the dispersal of the fungus?

The recorded data may also be important for understanding the recent spread of the species inland as collectors may transfer the spores with them.

## ﻿Methods

### ﻿List of localities in Europe

**Figure 1.**
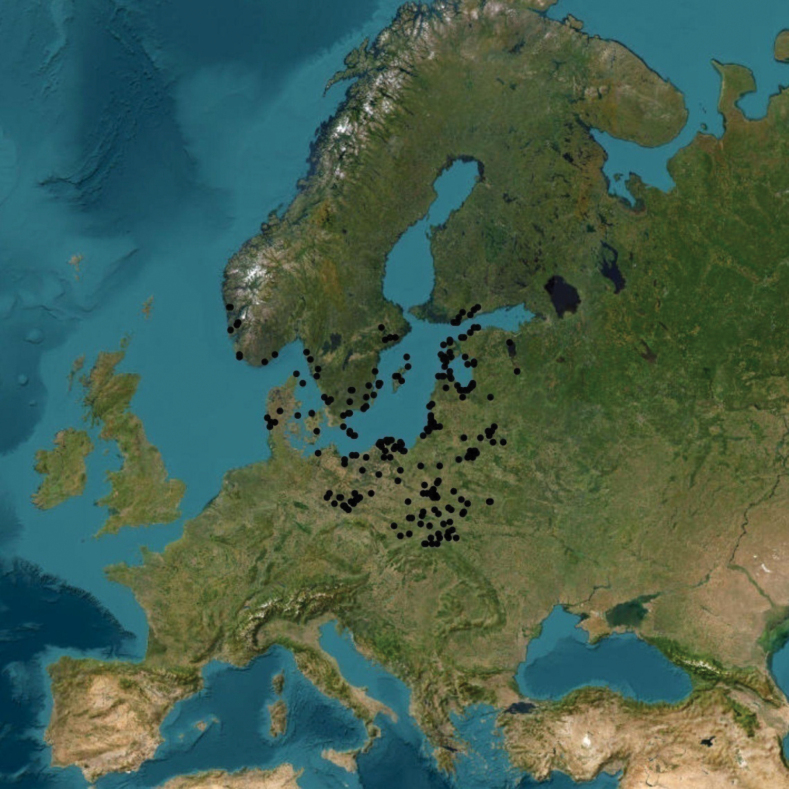
Distribution of *Aureoboletus projectellus* in Europe based on Banasiak et al. (2019b) and data gathered in open databases (GBIF 2025a), including the citizen science project “Distribution of *Aureoboletus projectellus* in Europe” (GBIF 2025b).

**Figure 2.**
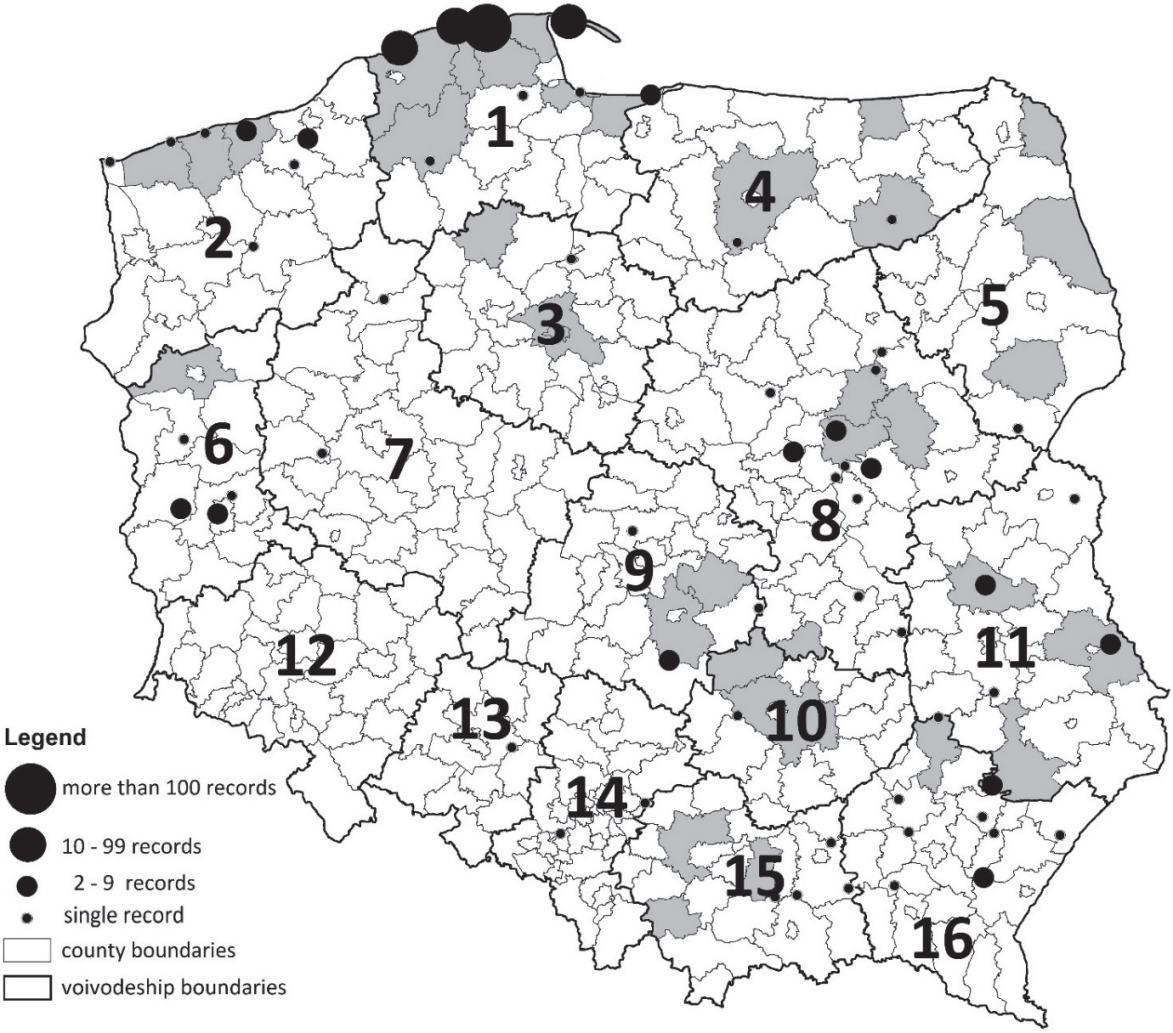
Distribution of *Aureoboletus projectellus* in Poland based on data gathered in this study (dots) and previous data (counties in grey) against a background presenting the county’s and voivodship’s administrative division (voivodships: 1 *Pomorskie*, region of the first introduction in Poland, 2 Zachodniopomorskie; 3 Kujawsko–pomorskie; 4 Warmińsko–mazurskie; 5 Podlaskie, 6 Lubuskie, 7 Wielkopolskie; 8 Mazowieckie; 9 Łódzkie; 10 Świętokrzyskie; 11 Lubelskie; 12 Dolnośląskie; 13 Opolskie; 14 Śląskie; 15 Małopolskie; 16 Podkarpackie).

Data on the current distribution of *A. projectellus* were gathered from the literature (Motiejūnaitė et al. 2011; Wrzosek et al. 2017; Banasiak et al. 2019b), herbaria, the Global Biodiversity Information Facility (GBIF 2025a, 2025b) and our own extensive field survey. Direct use of all GBIF records could be limited because of the autocorrelation of several closely located records. Thus, exclusively records for which the precise geographic coordinates were available were used in analyses. From a total of 447 locations of *A. projectellus* gathered in this study, the multiple species records from each grid cell (in the same 21.62 km^2^ equator) were removed. A final database included 325 occurrence data (Fig. 1). Both maps were generated in ArcGis 10.8 software powered by Esri. The distribution of the fungus in Poland was presented based ona map of counties (‘powiat’ of which there are 380 in Poland) and voivodships (16) as basic spatial units (Fig. 2).

### ﻿Questionnaire

In September 2019 we published an online questionnaire on the species, which was active till March 2024, and supplemented with a question about dishes made from the species in 2023. The survey was completely anonymous. We asked about general information (like gender, age, place of life, and place of birth) data specific to a given record (georeferences and date of observation), year of first observation, experience in the mushroom collection (number of collected species, number of known fungi), ethnomycological issues (name, that gatherers use, how they perceive with fruitbodies) and others. The full questionnaire is attached as a Suppl. material 1. The link to the questionnaire was published on social media, mainly on various Facebook fungi enthusiast forums. This yielded 165 answers (three questionnaires were excluded due to the nonserious nature of the responses). Additionally, interviews were done on the Baltic coast near Stilo, where the species was first observed in Poland, at a site popular among mushroom pickers (112 interviews). To confirm the correct identification of *A. projectellus* interviewers added the photo showing fruitbody in case of an online survey. For people interviewed in the forest, this confirmation was performed by naked eye, based on the content of their basket. Some go there specially to see or collect the species and they are advanced mushroom pickers. Altogether, 274 people provided meaningful information, either online or during field interviews, between 2019 and 2024.

### ﻿Data processing

To compare our data with existing data on the species distribution, we used our own first author’s (M.P.) unpublished data as well as available literature. Towards the end of our study, we encountered an international survey on the distribution of *A. projectellus* organized by Inaturalist (https://www.inaturalist.org/observations?project_id=aureoboletus-projectellus-spread-in-europe&subview=map&verifiable=any) but did not use it in statistical comparison, as some of our information suggested that some of our respondents later also filled in the Inaturalist questionnaire.

Spearman’s rank correlation was performed using PAST 4.03 (Hammer and Harper 2003) to evaluate the correlation between the collectors’ origin and the number of reports detected in a given regions. Because of the reverse relationship between the number of collectors and the number of records (collectors travelled specifically to the Pomorskie region knowing that the fungus occurs there, rather than encountering it incidentally and potentially spreading it further), we excluded Pomorskie from the analysis. We also present trends in the observed number of fungus records in Poland, expressed per time, distance, and cumulatively. We assessed the overall trend in the cumulative number of records over time by fitting both exponential and curvilinear functions. These estimators of introduction rate are among the most long-standing and frequently utilised indicators of biological invasions. (Liebhold and Tobin 2008; Tittensor et al. 2014; Seebens et al. 2017). We assumed that fungus was first reported in Europe in 2007 (Motiejūnaitė et al. 2011).

The anonymized data matrix was deposited in the Scientific Data Repository of Rzeszów University (see Data Availability section).

## ﻿Results

**Figure 3.**
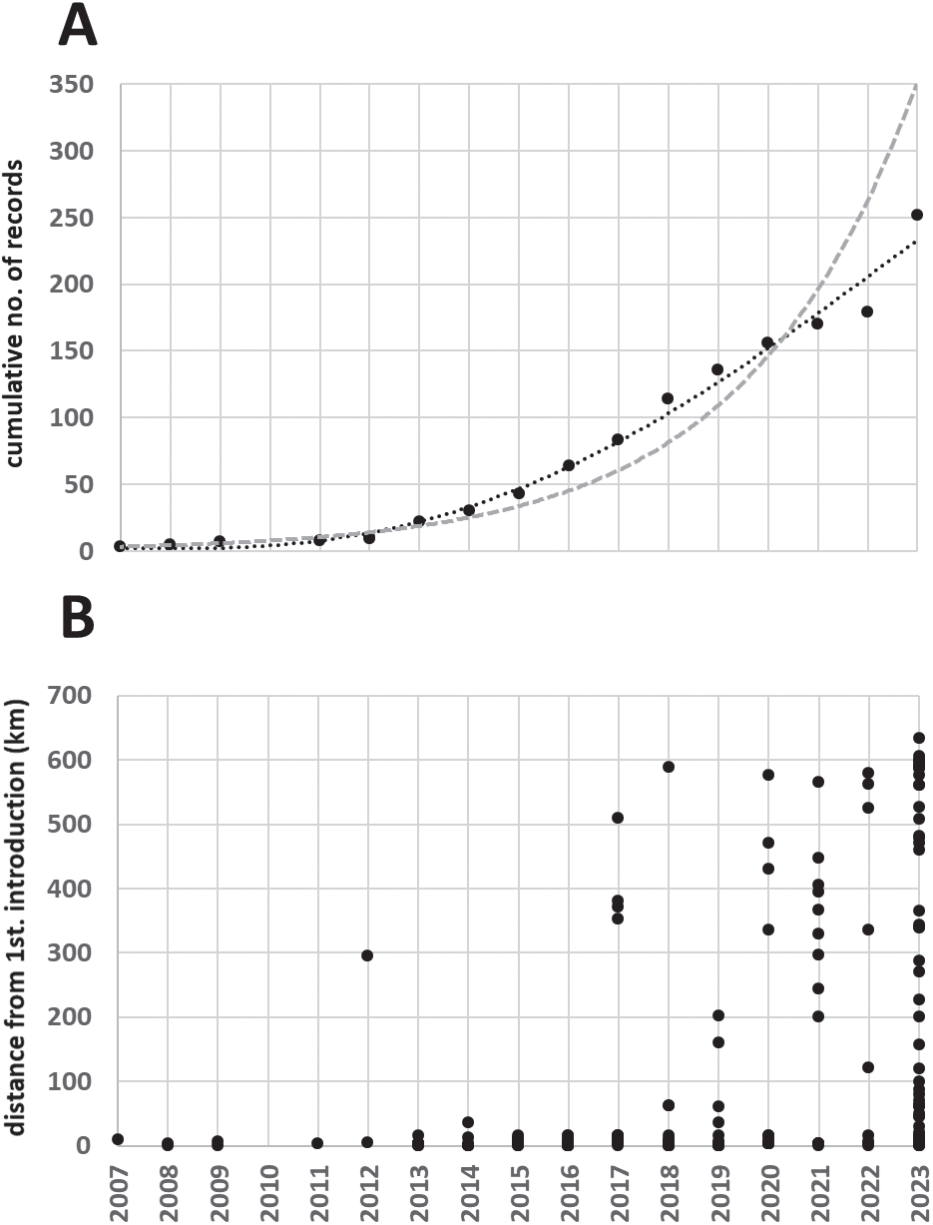
The trend in the cumulative number of records of *Aureoboletus projectellus* reflected by exponential (grey line) and curvilinear (black line) functions, since its first introduction in Europe in 2007 to 2023 (A), and the distance between the site of its first observation in Poland (Pomorskie voivodship, Stilo lighthouse near Sasino village) and the closest settlements, where the fungus was detected (B), (N = 252 represents the total number of interviews, excluding 22 reports from the site of first introduction between 2007 and 2023).

The majority of records of the species come from the region of its first report in Poland. In 205 out of 274 cases, the mushroom was gathered in the Pomorskie region. Only 53% of people collecting the fungus in the Pomorskie region came from the area. The other part were people who visited primarily to gather the fungus or were tourists and had encountered it on holiday (Fig. 2). Following its initial discovery in Poland, the fungus was collected almost exclusively along the coast (Fig. 3A). Over time, especially in recent years, the number of collectors has increased significantly (Fig. 3B). Data from an online questionnaire revealed 56 new localities of the fungus across Poland, including in 28 counties where no previous records had been reported. Notably, 80% of respondents were aware that the species is new and potentially invasive, although 19% were not.

**Figure 4.**
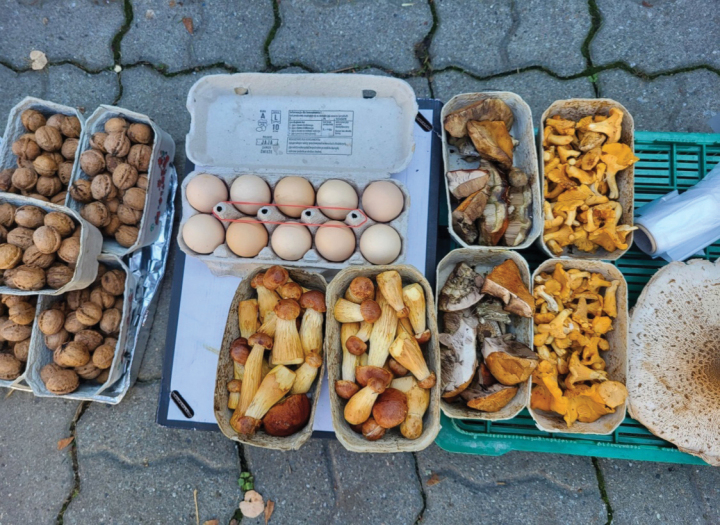
*Aureoboletus projectellus* sold in the local fungi market in Rzeszów in south-eastern Poland as a sought-after delicacy.

The distance between new collection sites and the original locality has increased over time (Fig. 3B). Until 2016, all observations of *A. projectellus* were confined to the Pomorskie region (no. 1, Fig. 2), with the exception of a single inland record in 2012. Since 2017 the fungus began to appear at locations increasingly distant from the original site of introduction (Fig. 3B). The distribution of records is uneven, and we found no correlation (Spearman’s rank), between the collectors’ origin and the number of reports detected in a given region (-0.08; p = 0.76). While the species is not collected in SW Poland, in Lower Silesia (Dolnośląskie; 12 Fig. 2), there is definitely a cluster of its localities in the neighbouring Lubuskie region further north. It has also not been reported from the Podlaskie region. Several localities of collection were reported from central Poland around the capital, Warsaw, where large population density is accompanied by extensive pine forests. However, what is most interesting is the large number of reported localities in SE Poland, in the lowland and sandy part of Podkarpackie region, where extensive pine forests can be found north and north-east from its capital, Rzeszów. This area is very far from the original invasion site on the coast. Nowadays the mushroom is even sold there in Rzeszów’s main vegetable market (Fig. 4). Interviewed sellers collected it between Lublin and Rzeszów.

Altogether, 52 local names of the species were recorded, with 26 mentioned by at least two people (Table 1). The different variants are usually formed using the word ‘American’, ‘heather’, ‘slender’ or ‘golden’ (Table 1, Suppl. material 2: table S1). The dominant versions are ‘American’ and ‘heather’– the former describes the species’ geographic origin, the latter its habitat in Poland, where it grows in pine woods with *Calluna vulgaris* (heather). The most commonly applied names are ‘borowik amerykański’, ‘borowik wrzosowy’, ‘amerykaniec’, ‘borowik wysmukły’ (the previous official vernacular name), ‘amerykanin’, ‘złotak’ and ‘złotoborowik ­wysmukły’ (the current official vernacular name).

**Table 1.** Names used for *A. projectellus* in Poland.

Polish local name	English translation	N = 345
borowik amerykański	American bolete	116
borowik wrzosowy	heather bolete	32
amerykaniec	The American	26
amerykan	The American	20
borowik wysmukły	slender bolete	15
amerykanin	American	13
złotak	golden	13
złotoborowik wysmukły	golden slender bolete	11
amerykański	American	10
prawdziwek amerykański	American bolete	9
złotoborowik	golden bolete	6
borowik wyniosły	slender bolete	5
borowik nadmorski	seaside bolete	4
prawdziwek	bolete	4
wrzosak	heathery	4
wrzosowy	heathery	4
złotak wysmukły	golden slender	4
borowik	bolete	3
prawdziwek wrzosowy	heather bolete	3
złotoborowik amerykański	golden American bolete	3
borowik sosnowy	pine bolete	2
borowik wrzosowaty	heather bolete	2
kanadyjski	Canadian	2
sasiniok	pine (sosna) – (combined with Sasino, village name)	2
złotak wyniosły	elevated golden	2
złoty borowik	golden bolete	2

The mushroom is perceived, both by our interviewees and by fungi collectors on social media forums, as tasty, fragrant and as possessing an interesting, firm texture. It is used similarly to other boletes, mainly in sauces, soups and pickles. It is also dried for further use (Suppl. material 2: table S2). Most often it is stewed or fried, used in soups, or pickled with vinegar or spices. It has started being served on Christmas Eve, the most conservative and ritual meal throughout the year, which usually includes only ancient Slavic traditional ingredients (flour, poppy seeds, honey, cabbage etc.), with mushrooms being a must (Referowska-Chodak 2015).

Out of 269 respondents who answered meaningfully, the most common answer to the question on preservation techniques was drying (61% respondents), then pickling (44%), freezing (61%) and consuming fresh, after processing (26%). Only one respondent mentioned lacto-fermenting with salt.

## ﻿Discussion

Recently, citizen science has been successfully applied in a wide range of studies on the biology and biogeography of plants (Puchałka et al. 2023; Aavik et al. 2025) and animals (Mori et al. 2019; Pocock et al. 2024). However, projects focusing on fungi remain relatively rare (Heilmann-Clausen et al. 2019; Kujawska et al. 2021; Pietras et al. 2021). Unlike most citizen science research, which relies on data from open-access repositories such as GBIF, our study is the first to employ an active data collection approach through an ethnomycological survey. Thus, the paper presents a unique case of an exotic fungus appearing on a new continent and quickly becoming a widely discussed article of food.

### ﻿Biogeography

The first documented record of *A. projectellus* in Europe was from the Curonian Spit in Lithuania in 2007 (Motiejūnaitė et al. 2011). The authors suggested that fruitbodies of the fungus may have been observed in this region as early as the 1980s; however, this claim cannot be substantiated with existing literature or available data. In Poland, the earliest record dates to before 2011 (Wrzosek et al. 2017), as confirmed in interviews gathered in this study. According to ethnomycological survey data, the fungus has been present in northern Poland since the late 1980s. Nonetheless, its earlier presence in Poland is not supported by long-term monitoring of mycobiota in the Pomeranian region (Stasińska and Sotek 2003; Stasińska 2005). Therefore, we consider 2007 as the potential starting point of the species’ expansion in Poland. Since then, the distribution range of the fungus has steadily grown, now extending over 600 km inland and encompassing almost all voivodships. Previously, the species had been recorded in 32 out of 380 counties of Poland. Our questionnaire-based survey confirms its presence in 60 counties. The fungus had previously been reported in 20 of these. In addition, this study revealed 56 new locations of the fungus in Poland, primarily in the southeastern part of the country.

The expansion pattern of *A. projectellus* generated based on ethnomycological interviews (Fig. 3A) corresponds to that of a typical invasion curve, illustrating the phases (Blackburn et al. 2011) and stages (Heger and Treplof 2003) of an alien organism’s invasion. During the initial stage (since its introduction to 2012), the fungus was recorded in only a few locations, all situated closely to the Baltic seashore (Wrzosek et al. 2018). In 2012, *A. projectellus* was reported for the first time in central Poland. The trend in the cumulative number of records over time, reflected by both exponential and curvilinear functions, indicates a rapid increase in fungus reports (Fig. 3A). Since 2017, the species has entered a rapid expansion phase, characterized by a sharp increase in population size and wide dispersal. Numerous new occurrences were reported in southeastern Poland – over 500 km from the original introduction site (Fig. 3B). The last year of interviews, 2023, showed the highest number of records, which increased in north, central and south Poland (up to 100 km, 100–400 km, and beyond 500 km, respectively, Fig. 3B). The cumulative curve of *A. projectellus* records (Fig. 3A) has not yet plateaued, suggesting that the species’ range is still expanding. This ongoing spread likely reflects continued colonization of new areas, with the fungus successfully dispersing, establishing, and reproducing across a broad geographic range, including sites at both short and long distances from the original introduction point. Based on the framework proposed by Blackburn et al. (2011), the observed expansion pattern of *A. projectellus* supports its classification as an invasive fungal species.

### ﻿Ethnomycology

Out of several alien plant species that have found culinary use in Poland, *A. projectellus* is the first exotic fungus to be widely eaten. As far as plants are concerned, the prime example of an invasive species used for food is *Robinia pseudoacacia*, introduced to Poland at the turn of the 18^th^ and 19^th^ century, whose flowers have been popularly made into fritters (Łuczaj 2010). Some other invasive plants, such as *Galinsoga* spp. (Diaz-Belancourt et al. 1999) eaten in its native South America and *Impatiens glandulfiera* eaten in its native Kashmir (Srivastava 1988), have only recently become an object of culinary interest in foraging and herbalist circles due to the popularity of wild food in general (Łuczaj, personal observations).

Intercontinental invasions of other *Boletaceae* and truffles have not yet been documented, either in biogeographical or ethnobiological terms (Hall et al. 1998; Murat et al. 2008; Dickie et al. 2016). Only the occurrence of these species has been recorded, however without deeper insight into how the appearance of new edible species influenced the culinary culture. Mushroom users tend to be conservative in their choice of species (Łuczaj and Nieroda 2014), however the *A. projectellus* was quickly ‘accepted’ as edible. What made it so easy? First of all, their ‘classic’ shape with spongy hymenophores (*Boletaceae* and *Suillaceae*) is regarded as relatively safe in Poland, i.e. either edible or, if bitter, slightly toxic. Education on edible mushrooms often starts from this piece of information (Łuczaj and Nieroda 2014). Once the most daring mushroom experts established that the species is not bitter and is actually tasty, it was quickly classified as edible. Also its colour resembles the edible *Boletus edulis*, *Suillus* spp. *Imleria badia* and *Xerocomellus* spp., the most commonly collected fungi in Poland. Thus, the new species was considered a variation in a folk genus of several edible species with common characteristics (for explanation of ‘folk genus’ see Berlin (1992)). Unfortunately, it cannot be legally sold as, according to European Union regulations, it is a ‘novel food’, not used in the E.U. before 1997, due to which its introduction to EU markets would require more complicated procedures and tests (see Novel Food website).

An interesting phenomenon mentioned by one of the respondents is the fact that internet mushroom forums require members to use official Polish names and ban them for using local spontaneous names. Thus, the spontaneously created common name ‘heather bolete’ may be replaced by ‘golden bolete’, as the species has now been moved to the genus *Aureoboletus*, meaning ‘golden bolete’.

*Aureoboletus projectellus* is already sold in markets even as far as Rzeszów in south-eastern Poland (Ł.Ł.– personal observations; Fig. 4). This happens even though the species is not on the official list of fungi species that are allowed to be sold to the public (Kotowski 2016; Minister of Health of the Republic of Poland 2022). The list is updated every few years, and the question if this species should be sold in markets will divide mycofans. On the one hand, it belongs to the *Boletaceae* group, prized in Polish cuisine and sought-after as food. On the other, its sales in different places may facilitate the spread of spores, when we still do not know how its presence might affect fungal communities.

### ﻿Do collectors spread *A. projectellus*?

The collection of *A. projectellus* has become an element of ‘foraging tourism’. This is a newly emerging type of tourism where visitors not only seek new culinary ingredients but gather them themselves (de Jong and Varley 2018). Overall, the large number of questionnaires submitted from different parts of the country demonstrates the great power of citizen science in ecological and ethnobiological research. Citizen science programs using internet questionnaires and apps have previously been used in biogeographical research (e.g. Devictor et al. 2010; Kumar et al. 2019) and most studies dealing with the biogeography of invasive fungi in Europe have been made thanks to information gathered in public databases, like GBIF, or iNaturalist (Pietras et al. 2018, 2021). However, they were only recently introduced in ethnobiology (Vandebroek and Albuquerque 2024). Questionnaires sent to citizens were issued in a number of eastern European countries in the 19^th^ century and brought and immense amount of data (Łuczaj and Köhler 2014; Köhler 2015; Kalle et al. 2022) about plant uses. The Polish Ethnographic Atlas also used questionnaires sent by local data collectors when collecting data on the use of wild edible plants and fungi, as well as medicinal plants, since 1948 (Łuczaj 2010). This study follows similar approach by gathering part of the data in the form of an online questionnaire.

Several management strategies have been proposed to mitigate the spread of ectomycorrhizal fungi into new areas. One such strategy to slow the invasion of edible fungi is reducing human-related dispersal (Dickie et al. 2016). Previous studies have shown that climatic conditions in Europe are favourable to *A. projectellus*, making its spread nearly inevitable (Banasiak et al. 2019a), furthermore, restricting the collection of fungi appears unrealistic in the context of Polish cultural and culinary traditions. Any attempt to limit fungal foraging in Poland could also intensify conflicts related to future invasive species management. The collected data show that a great number of people from different regions of Poland have visited the large *A. projectellus* sites on the Baltic coast, which may have been an important vector of the spread of the fungus in inland Poland (Table 2). However, we found no correlation (Spearman’s rank) between the collectors’ origin and the number of reports detected in a given regions. For example, the fungus is now often reported in the Podkarpackie region, where no people who had collected the fungus in the north of Poland were encountered. Conversely, the questionnaire yielded several people from Śląskie and Dolnośląskie who collected the fungus in the north, but hardly any localities of this fungus are known from those regions. This shows that even if spreading the spores by mushroom-pickers may be an additional facilitating factor, the main vector in the species’ invasion may be an animal vector or wind.

**Table 2.** Origin of collectors of *A. projectellus* in the region of first appearance (Pomorskie region) compared to the number of reports of collecting *A. projectellus* in different regions.

Region (województwo)	Origin of collectors of A. projectellus in Pomorskie region, the region of first appearance	No. of reports of collecting A. projectellus in different regions
Pomorskie	109	205
Dolnośląskie	22	0
Kujawsko-pomorskie	4	0
Lubelskie	3	8
Lubuskie	5	6
Łódzkie	8	4
Małopolskie	9	5
Mazowieckie	17	13
Opolskie	3	1
Podkarpackie	0	12
Podlaskie	0	1
Śląskie	15	2
Świętokrzyskie	1	1
Warmińsko-mazurskie	1	3
Wielkopolskie	6	1
Zachodnio-pomorskie	1	12

The decision on whether to further advertise the species’ edibility should depend upon the results of studies investigating the impact of *A. projectellus* on native fungal communities. If it is very detrimental to them, people should be educated and discouraged from eating and collecting in order to slow down its spread, as commerce, especially across long distances, dramatically increases the spread of invasive organisms (Lenda et al. 2014). Otherwise, it could be added to the list of commercially utilized mushrooms as it is sold in some markets anyway. Unfortunately, currently not enough data is available to assess the impact of *Aureoboletus projectellus* on the local mycobiota, due to which the species is better referred to as exotic instead of invasive.

## ﻿Conclusions

1. Our research provided early data on the gathering practices of *A. projectellus* in Poland.
2. The mushroom is already commonly gathered and has been given several vernacular names by mushroom pickers.
3. The species is treated as a valuable food item, with a taste comparable to ordinary boletes, and has been included in the Polish culinary culture in recipes similar to those for other boletes.
4. No correlation was found between the origin of mushroom collectors along the Baltic Sea coast and the inland localities where the fungus occurs. Thus, we found no evidence that mushroom collectors are an important vector for spreading this alien species in Poland.
5. Further spread of both the species and its utilization should be monitored in Europe.

## ﻿Additional information

### Use of AI

No use of AI was reported.

### Author ORCIDs

Marcin Pietras https://orcid.org/0000-0002-2692-0257

Dominika Robak https://orcid.org/0000-0002-9356-0642

Magdalena Terlecka https://orcid.org/0000-0003-1200-2612

Łukasz Łuczaj https://orcid.org/0000-0001-5272-4826

### Data availability

The anonymized data matrix was deposited in the Scientific Data Repository of Rzeszów University (Repozytorium Danych Badawczych Uniwersytetu Rzeszowskiego website, see https://rdb.ur.edu.pl/items/5d6de1fd-32df-432c-ba2e-dfe1073ef837).

## Supplementary materials

Supplementary material 1 The interview used in this study addressed to the collectors of the *Aureoboletus projectellus* advertised in fungi forums with the picture of the mushroom Data type docx https://binary.pensoft.net/file/1437638This dataset is made available under the Open Database License (http://opendatacommons.org/licenses/odbl/1.0/). The Open Database License (ODbL) is a license agreement intended to allow users to freely share, modify, and use this Dataset while maintaining this same freedom for others, provided that the original source and author(s) are credited.Łukasz Łuczaj

Supplementary material 2 Supplementary tables S1, S2 Data type pdf Explanation note **table S1**: Semantics of local names of *A. projectellus* in Poland; **table S2**: Types of dishes made using *A. projectellus*. https://binary.pensoft.net/file/1437639This dataset is made available under the Open Database License (http://opendatacommons.org/licenses/odbl/1.0/). The Open Database License (ODbL) is a license agreement intended to allow users to freely share, modify, and use this Dataset while maintaining this same freedom for others, provided that the original source and author(s) are credited.Łukasz Łuczaj
