# Supplementary material for: ﻿Does a citizen science project describe the biogeography of exotic Aureoboletus projectellus in Poland? An ethnomycological survey
Source: IMA Fungus. 2025 Oct 14;16:e166407. doi: 10.3897/imafungus.16.166407 (PMC12541463; doi:10.3897/imafungus.16.166407)
Supplement: Supplementary material 1 — The interview used in this study addressed to the collectors of the Aureoboletus projectellus advertised in fungi forums with the picture of the mushroom [file imafungus-16-e166407-s001.docx]

**Appendix**

The interview used in this study addressed to the collectors of the *Aureoboletus projectellus* advertised in fungi forums with the picture of the mushroom.

1. Age

2. Gender

3. Where was the mushroom collected (specify the closest settlement)?

4. Where do you live (including county)?

5. Where were you raised (including county)?

6. Since which year have you collected the fungus?

7. Do you ever travel only to collect this species?

8. What name do you apply to this taxon?

9. When visiting the forest, what is your main aim?

10. In what places do the fungus occur?

11. How did you find out about them?

12. What are the differences between the fungus and other fungi?

13. How many specimens do you collect per day?

14. How are they stored? Fresh/dried/frozen/pasteurized/other?

15. Are you a skilled collector? (1-5)

16. Which species do you collect apart from *Aureoboletus projectellus*? Which of them most frequently?

17. Are you aware that *Aureoboletus projectellus* is a potentially invasive species?

18. List dishes made from this fungus.

19. Do you have a photo of the specimen ?

20. Comments
