# Supplementary material for: ﻿Does a citizen science project describe the biogeography of exotic Aureoboletus projectellus in Poland? An ethnomycological survey
Source: IMA Fungus. 2025 Oct 14;16:e166407. doi: 10.3897/imafungus.16.166407 (PMC12541463; doi:10.3897/imafungus.16.166407)
Supplement: Supplementary material 2 — Supplementary tables S1, S2 [file imafungus-16-e166407-s002.pdf]

Table S1. Semantics of local names of *A. projectellus* in Poland

Stem of name	English meaning	Frequency (n=345)
ameryk-	American	230
borowi-	bolete	175
wrzos-	heather	47
złot-, złoc-	golden	42
wysmuk-, smuk-	slim	32
prawdziw-	bolete	20
wynios-	elevated	7
sosn-	pine	5
nadmor-	seaside	4
kanadyjski	Canadian	3
chud-	thin	2
łebski	of Łeba (a town around which it is common)	2

Table. S2. Types of dishes made using *A. projectellus*.

Dish	Number of citations, *n*=66
Stewed for sauce	27
Soup	16
Pickled in vinegar (usually with spices) as a side-dish	15
Fried	11
Dumplings (pierogi)	5
Goulash	4
With scrambled eggs	4
Served in dishes on Christmas Eve	3
Pasta	3
Cabbage dishes (bigos)	3
Risotto	2
Toast	2
Pizza	2
